# Nonuniform subduction of the Indian crust beneath the Himalayas

**DOI:** 10.1038/s41598-017-12908-0

**Published:** 2017-10-02

**Authors:** Xiaoyu Guo, Wenhui Li, Rui Gao, Xiao Xu, Hongqiang Li, Xingfu Huang, Zhuo Ye, Zhanwu Lu, Simon L. Klemperer

**Affiliations:** 1 School of Earth Sciences and Engineering, Sun Yat-Sen University, Guangzhou, 510275 China; 2 Institute of Geology, Chinese Academy of Geological Sciences, Beijing, 100037 China; 3 Department of Geophysics, Stanford University, Stanford, California, 94305 USA

## Abstract

Himalayan tectonic activity is triggered by downward penetration of the Indian plate beneath the Asian plate. The subsurface geometry of this interaction has not been fully investigated. This study presents novel constraints on this geometry provided by two newly obtained, deep seismic reflection profiles. The profiles cover 100- and 60-km transects across the Yarlung-Zangbo suture of the Himalaya-Tibet orogen at c. 88°E. Both profiles show a crustal-scale outline of the subducting Indian crust. This outline clearly shows Indian understhrusting southern Tibet, but only to a limited degree. When combined with a third seismic reflection profile of the western Himalayas, the new profiles reveal progressive, eastward steepening and shortening in the horizontal advance of the subducting Indian crust.

## Introduction

**Figure 1:**
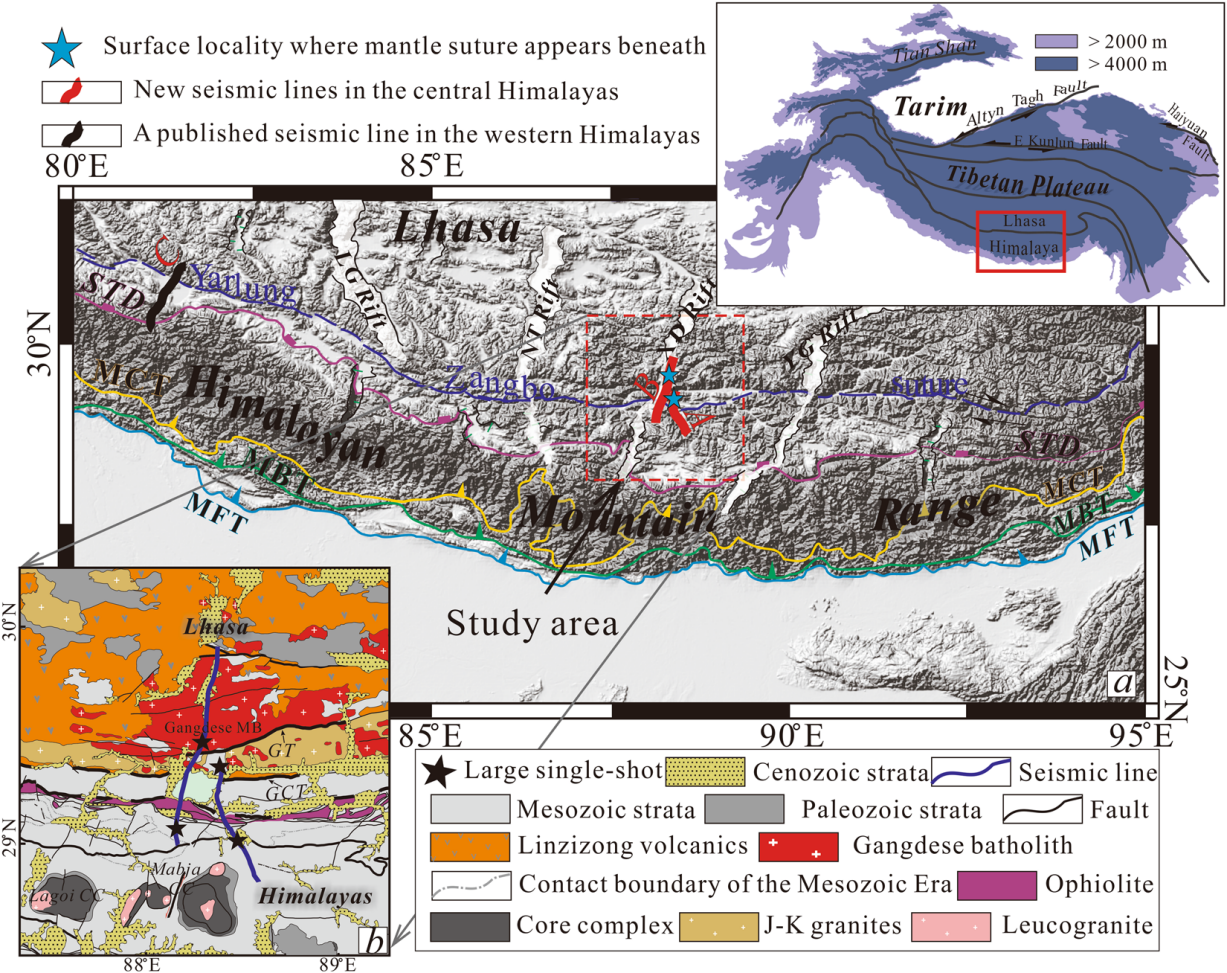
(**a**) Topographic map of southern Tibet and the Himalayan Mountain Range showing the location of two 60 km and 100 km seismic reflection profiles (lines A and B, respectively) interpreted here. Line C represents previous seismic transects covering the western Himalayas^24^. (**b**) Generalized geological map of the research area was modified after The Geology Map of China [1:1,000,000]. Black stars indicate the sites of four single shots with 2000 kg explosive sources. The inset shows the location of the research area (red box) in a larger regional context. TB: Tarim basin, TP: Tibetan Plateau, SCB: South China Block, IP: Indian Plate, KF: Kunlun fault, LG Rift: Longgar Rift, NT Rift: Nyima-Tingri Rift, XD Rift: Xainza-Dingjye Rift, YG Rift: Yadong-Gulu Rift, GCT: Great Counter Thrust, GT: Gangdese Thrust. Digital elevation data were downloaded from the website of http://srtm.sci.cgiar.org. Figures A and B, as well as the inset were drafted by X.G. and X.X. using the software of CorelDRAW X5.

The Himalayan-Tibetan Plateau is currently the world’s largest example of an active continent-continent collisional orogen. The plateau formed primarily due to the collision between the Indian and Eurasian tectonic plates after subduction and closure of the intervening Neotethyan Ocean in the past 55 Ma^1^. The plateau rises 4–5 km above sea level and spans 2900 km in an east-west direction (inset in Fig. 1). The plateau is scientifically significant due to its size, elevation and crustal thickness, which is twice the global average. Due to its position, this feature also exerts a strong influence on the regional climate of South Asia^2^. In addition, the plateau and its formation generate significant regional seismic activity, including the 2008 *Mw* 7.9 Wenchuan earthquake in eastern Tibet^3^ and the 2015 *Mw* 7.9 Gorkha earthquake (Nepal) in southern Himalayas^4^. These events caused numerous fatalities and significant property damages.

Over the past few decades, geoscientists have analyzed the India-Eurasia collision using a range of different geological and geophysical tools. This research has generated several different models for explaining the Tibetan Plateau’s unusual style of uplift^1,5–8^. Models attribute plateau construction to either a doubling of crustal thickness that occurred when the Indian crust had underthrust most of Tibet^5^ or to crustal shortening that did not require the presence of the Indian crust beneath the plateau^1^. The extent of the subducted Indian crust beneath the Tibetan Plateau is thus a critical assumption in models seeking to explain plateau history. Previous receiver function studies have provided unequivocal constraints on the northward extent of the downwelling Indian continental lithosphere^8–13^. Given the data resolution of these studies however, crustal geometry within the collision zone remains ambiguous. Multichannel seismic reflection profiling experiments in the 1990s recorded lithosphere-scale structure of the Himalayas^14–16^, but the exact geometry of the collisional suture remains poorly constrained. Previous interpretations have been based on single south-north cross-sections and assumed uniform subduction of the Indian crust. It is not certain however that the Indian crust does subduct in a uniform manner across the entire 2900 km-wide orogenic belt. Results from individual seismic profiles integrate consistently with local structural interpretations but have not provided consistent interpretations for crustal geometry at orogen-scale.

In this study, we present two recently acquired, high-resolution, deep seismic reflection profiles that span the Yarlung-Zangbo suture zone of the central Himalayas (Fig. 1a). Emerging data collection and processing methods enabled acquisition of high quality reflection images showing deep subsurface structure. The overall image clearly shows the geometry of the Indian crust beneath Tibet and critical spatial variation in its subducted/underthrust areas.

## Two new deep seismic reflection profiles across the Yarlung-Zangbo suture

The two profiles run 120 km in an N-S direction and overlap by ~40 km around the Yarlung-Zangbo suture (Fig. 1a). These transects therefore cover a swath of the Tethyan Himalaya to the south and a broad swath to the north that includes the west-northwest-trending Yarlung-Zangbo suture and Gangdese magmatic belt to the north (Fig. 1b). The Tethyan Himalaya in the study area consists of thick marine sedimentary and ophiolitic sequences that range from Precambrian to Cretaceous in age (Fig. 1b)^17^. The Mabja metamorphic core complex (Fig. 1b) near the southern terminus of the seismic profiles has been exposed at the surface by mid-crustal ductile extension initiated at ~35 Ma and lasting ~12–19 my^18^. The profiles interpreted in this report also span the Great Counter Thrust (GCT) and Gangdese Thrust (GT) (Fig. 1b)^19^. Structural field investigations show top-to-the north displacement along the GCT^18^. Age constraints suggest an early Miocene initiation (25-23 Ma)^18,20–22^ for the GCT. The GT bounds the southern margin of the Gangdese magmatic belt (Fig. 1b) and has been constrained at 27-23 Ma by ^40^Ar/^39^Ar methods^23^.

Data collection and processing steps followed those described in our previous seismic studies of the western Himalaya^24^. We deployed three types of explosive sources: 50 kg shots in single shot holes at 250 m intervals, 200 kg shots in pairs of shot holes at intervals of 1 km, and 2000 kg shots in clusters of 10 shot holes at 50 m depth over intervals of 50 km. Data were recorded by 600 receiver traces over a 60 s two-way travel time window. This experimental setup provided a nominal 60-fold common mid-points (CMPs) stacked section. Processing steps also used Kirchhoff pre-stack time migration (see Methods details).

**Figure 2:**
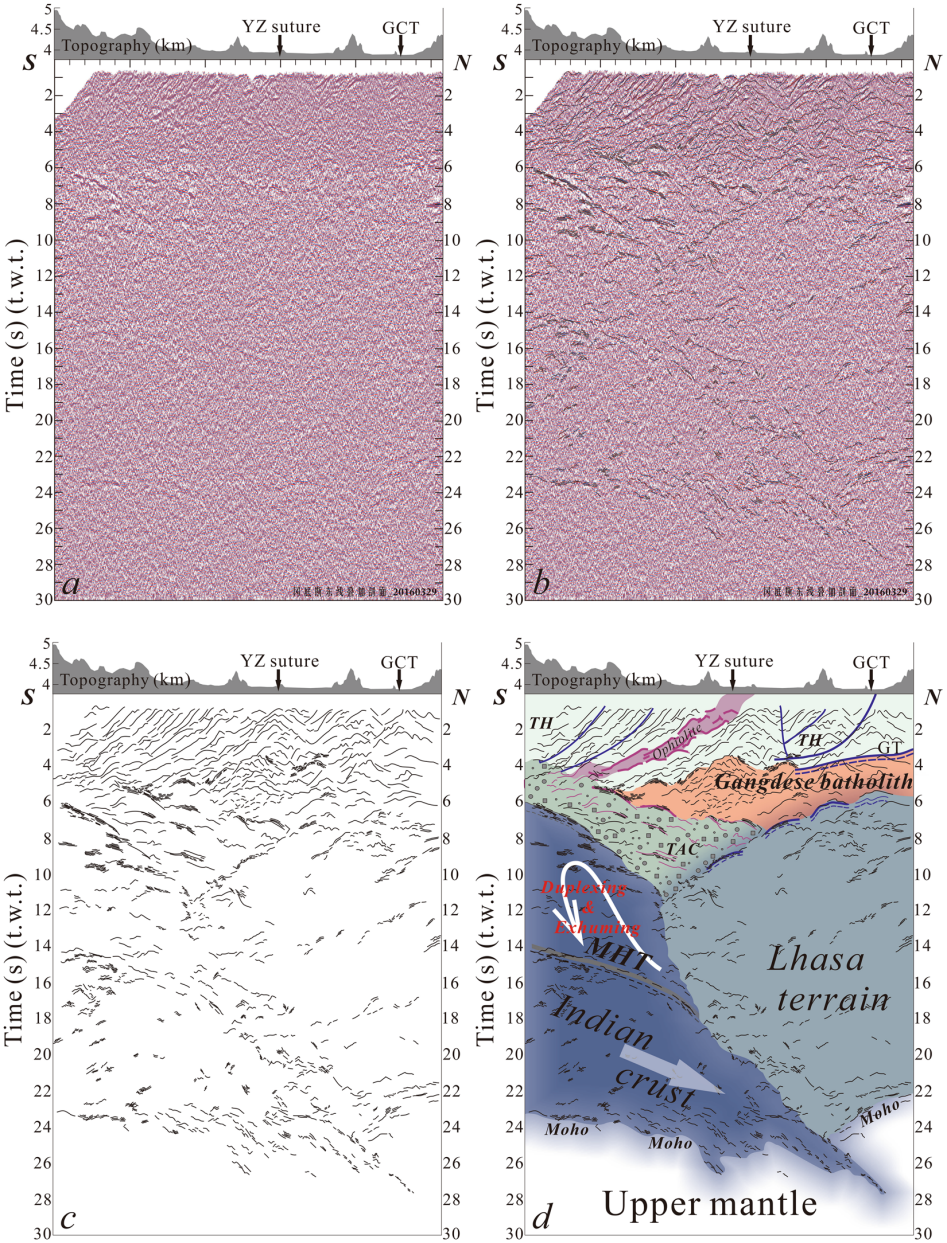
Uninterpreted deep seismic reflection profile (no vertical exaggeration) of the east line (**a**). See Fig. 1 for surface trace of transect (Labeled in A). Superimposed line drawings of high-amplitude reflectors from the seismic section (**b**) and composite line drawings showing major features (**c**). Structural interpretation integrating surface geology and reflection patterns (**d**). The high-amplitude reflectors in purple represent fragments of ophiolitic mélange from the Yarlung-Zangbo suture dislocated during crustal-scale duplexing of the subducted Indian crust. GCT: Great Counter Thrust, GT: Gangdese Thrust, YZ: Yarlung-Zangbo, TH: Tethyan Himalaya, TAC: Tethyan accretionary complex, MHT: Main Himalayan Thrust. Figure 2a contains raw seismic reflection data that were collected in the field. Superimposed line drawings in Fig. 2b and c, as well as structural interpretation in Fig. 2d, were drafted by X.G. and X.X. using the software of CorelDRAW X5.

**Figure 3:**
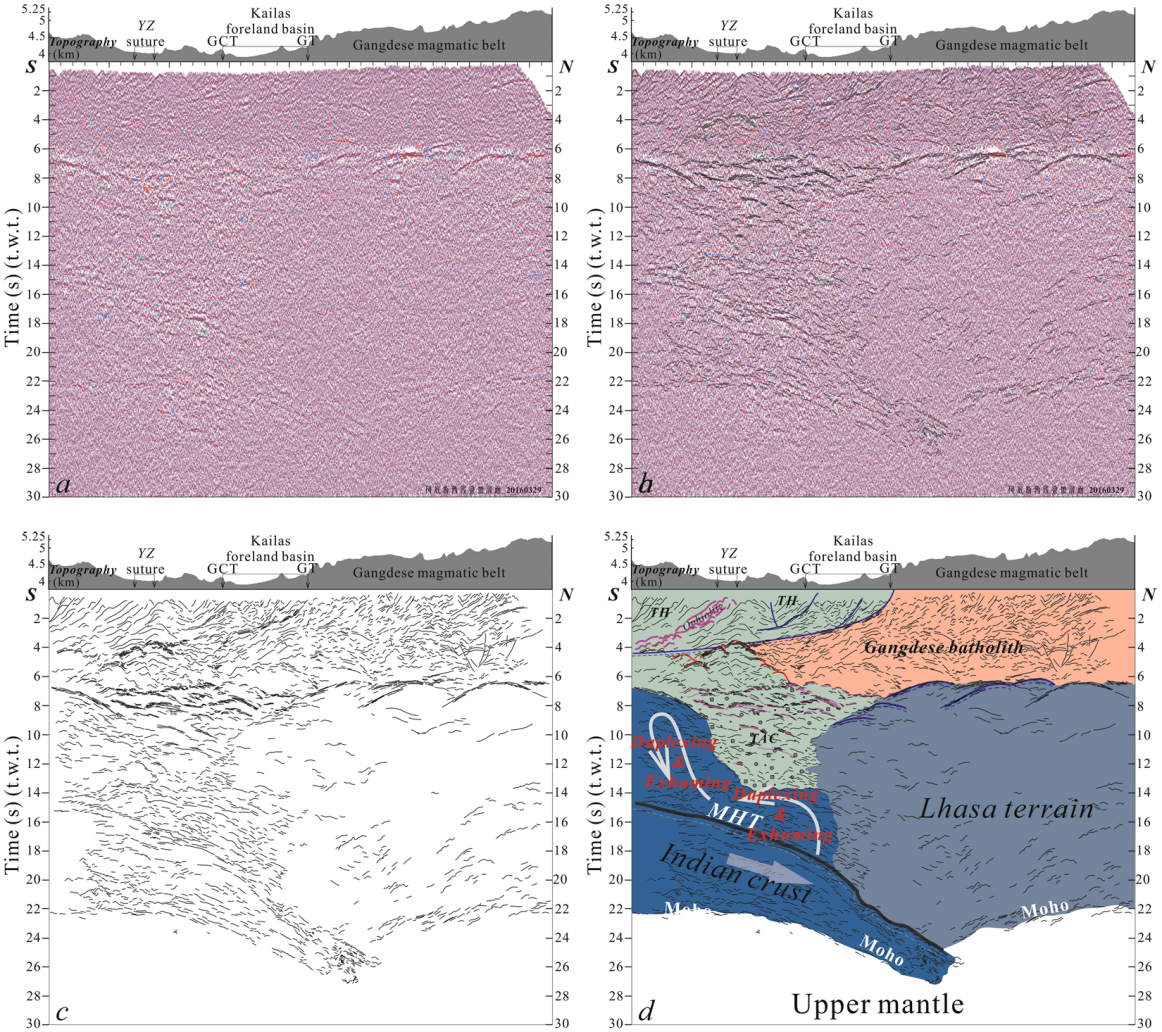
Uninterpreted deep seismic reflection profile (no vertical exaggeration) of the west line (**a**). See Fig. 1 for surface trace of transect (labeled in B). Superimposed line drawings of the high-amplitude reflectors from the seismic section (**b**) and composite line drawings showing major features (**c**). Structural interpretation integrating surface geology and reflection patterns (**d**). The high-amplitude reflectors in purple represent fragments of ophiolitic mélange from the Yarlung-Zangbo suture dislocated during crustal-scale duplexing of the subducted Indian crust. GCT: Great Counter Thrust, YZ: Yarlung-Zangbo, TH: Tethyan Himalaya, TAC: Tethyan accretionary complex, MHT: Main Himalayan Thrust. Figure 3a contains raw seismic reflection data that were collected in the field. Superimposed line drawings in Fig. 3b and c, as well as structural interpretation in Fig. 3d, were drafted by X.G. and X.X. using the software of CorelDRAW X 5.

Figures 2a and 3a show the near-vertical, uninterpreted seismic reflection images for the east and west lines, respectively. These extend to about 30 s two-way time (t.w.t.) (Figs 2a and 3a) or ~90 km depth (assuming an average crustal velocity of 6 km/s). To visualize overall structure of the area imaged, we observed high-amplitude reflections in the seismic transects (Figs 2b and 3b) and constructed composite line drawings for the east and west lines, shown in Figs 2c and 3c (respectively). Figures 2d and 3d present structural interpretations that integrate seismic reflection data and previous geological interpretations for the east and west lines, respectively.

**Figure 4:**
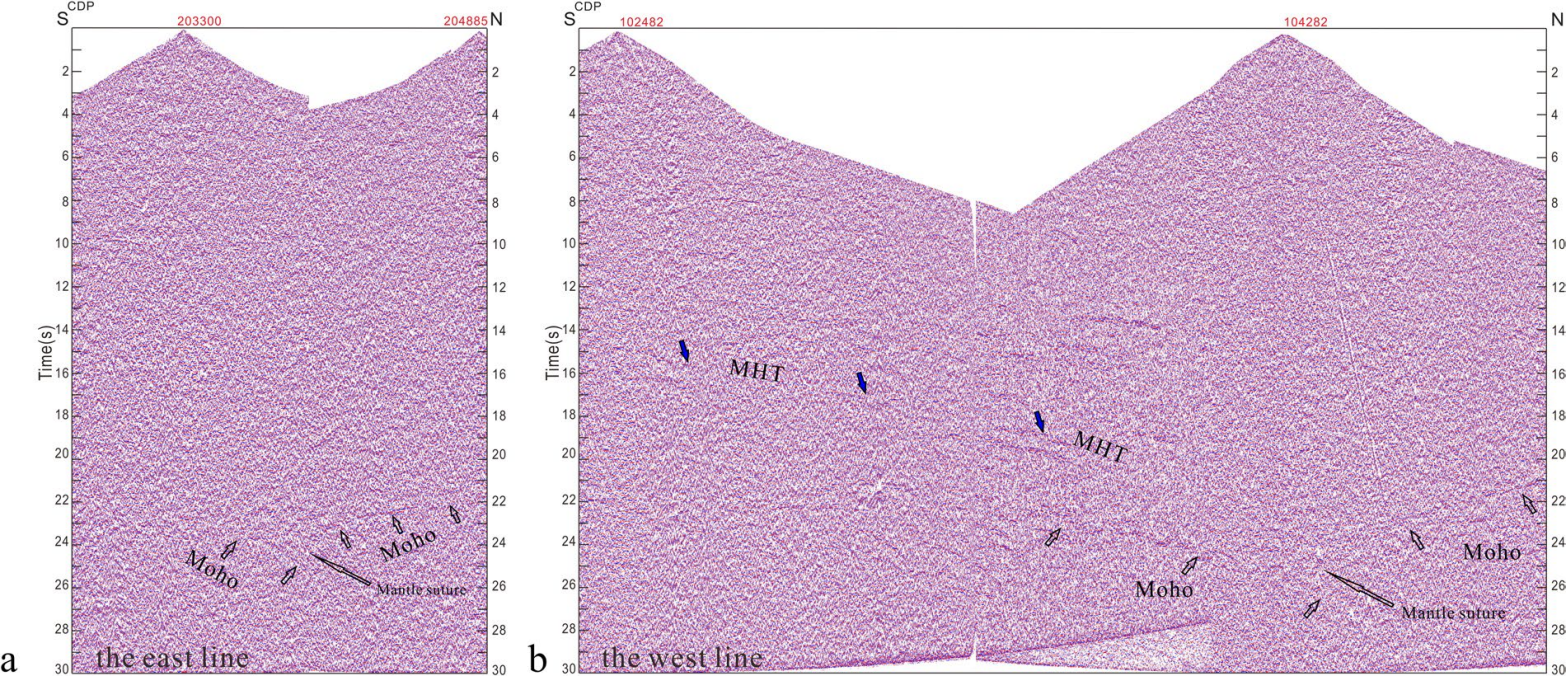
Single-shot section from the 2000 kg explosive source along the east (**a**) and west (**b**) lines. Mantle suture appears beneath a crustal section thickened to twice its global average thickness. Blue stars in Fig. 1a indicate individual surface localities where mantle suture appears beneath the Lhasa terrain. Black stars in Fig. 1b show shot sites along the two seismic reflection lines.

The two seismic profiles clearly outline the structural geometry of the collision zone (Figs 2c and 3c). The base of the lower crust in both seismic transects appears as a zone of high-amplitude reflectors at depths of ~22–27 s (t.w.t). Given the ~66–81 km depth range (assuming an average crustal velocity of 6 km/s), we interpreted this feature as the crust-mantle boundary (Moho). Both seismic profiles show a crustal thickness of two times the global average. The profiles also show a converging geometry indicating an offset structure in the Moho (Figs 2c,d and 3c,d). This structure (interpreted as a mantle suture) appears in single shot, 2000 kg source datasets from both east and west lines (Fig. 4a,b, respectively; 2000 kg shots shown as black stars in Fig. 1b).

Upper crustal areas of seismic images show a series of steep, south-dipping reflectors truncated at their base by two sets of reflections exhibiting a ramp-flat geometry (Figs 2c and 3c). In accordance with previous studies of surface geological features, we interpret these basal, southerly-dipping reflections (0–4 s, t.w.t) as corresponding to the GCT that represents thrusting emplacement of the Tethyan Himalayas over the Kailas succession^17^ (Figs 2d and 3d) and the GT that marks the southern edge of the Gangdese belt^17^ (Fig. 2d). The GCT and GT appear to displace the base of the Yarlung-Zangbo suture complex in a low-angle thrust giving each element a southerly dip (Figs 2d and 3d). It is noteworthy that, instead of being top to the south, the whole-scale geometry of the GT in the seismic transect appears top to the north, which is opposite to previous studies from surface geological investigations^23^. In addition, surface structural investigations have documented top-to-the north displacement bounding the ophiolitic mélange^17^, which also appeared in the INDEPTH seismic reflection profile across the Yarlung-Zangbo suture zone^16^. This observation runs contrary to the assumption that faults bordering the exposed suture zone trend parallel to the direction of subduction. The bivergent structure appearing in the upper crust indicates progressive thickening within the accretionary prism and the area to the south. The suture zone within the upper crust’s accretionary prism thus steepened and developed into a retrowedge with southward truncation of the GCT and Gt at its base.

Along the southern segment of the seismic transect, a group of prominent reflectors appear at depths of 14–15 s (t.w.t.) and extend continuously downward to a depth of 28 s (t.w.t.) (Figs 2a and 3a, see additional review material for higher-resolution seismic images). Comparison with features identified by previous seismic reflection and wide-angle studies of the southern Himalayan Mountain Range^12,14,25^ indicate that laterally extensive reflectors at 14–15 s (t.w.t.) depth represent the Main Himalayan Thrust (MHT). The Main Central Thrust (MCT), the Main Boundary Thrust (MBT) and the Main Frontal Thrust (MFT) are supposed to branch on the MHT, a major regional detachment fault^12,14,25^. An additional set of high amplitude reflectors (bright-spot) appear just above the MHT at depths of 6–15 s (t.w.t.) in the Tethyan accretionary complex (TAC) (Figs 2c and 3c). These sub-horizontal reflectors terminate to the north against a set of south dipping “bright-spot” reflections. This latter feature differs from the overlying crust and accretionary prism to the north. Given the well-documented crustal-scale duplexing that has transferred material from the lower to the upper plate in the western Himalayas^24^, we interpret this anomalous zone (bright-spot) as consisting of fragments of ophiolitic mélange from the Yarlung-Zangbo suture (Figs 2d and 3d) dislocated during crustal-scale duplexing of the subducted Indian crust along the MHT at its base^24^ (Figs 2d and 3d).

In northerly areas of both seismic transects (Figs 2a and 3a), areas beneath 8 s (t.w.t) in Fig. 2a and beneath 6 s (t.w.t) in Fig. 3a appears as a zone having only a few short-wavelength south dipping reflectors. The basal geometry of the Moho and orientation of its offsets indicate that this zone represents the crystalline basement of the Lhasa terrain undergoing intense collisional compression. High temperatures therein may eliminate long-wavelength converters.

The continuous trace of the MHT and prominent Moho reflections (Figs 2c and 3c, see additional review material for higher-resolution seismic images) highlight the geometry of the subducting Indian crust (Figs 2 and 3). Images clearly show the limited extent of Indian crust subduction beneath the Lhasa terrain. Figure 1a (blue stars) shows surface projections of Moho offsets (mantle suture). The subducting Indian crust extends beneath the Gangdese batholith along the western profile but only advances to a limited extent beyond the northerly Yarlung-Zangbo suture zone along the eastern one. The western profile thus shows greater horizontal advance for the subducting Indian crust than that indicated by the eastern profile (Fig. 1a). Leading edge of the subducting Indian crust within the western profile exhibits a dip angle of about 27°NE (Fig. 2d), while the eastern profile indicates a dip angle of about 45°NE (Fig. 3d). Previous seismic reflection studies of the western Himalaya (solid black line C in Fig. 1a) detected relatively flat Moho reflections and no offset related to continent-continent collision beneath the dominant collision zone^24^. The overall crustal geometry imaged by these seismic reflection profiles (seismic lines A, B and C in Fig. 1a) affirms lateral variation along the subductive margin and indicates progressive eastward steepening of the down-going Indian crust.

## Discussion

**Figure 5:**
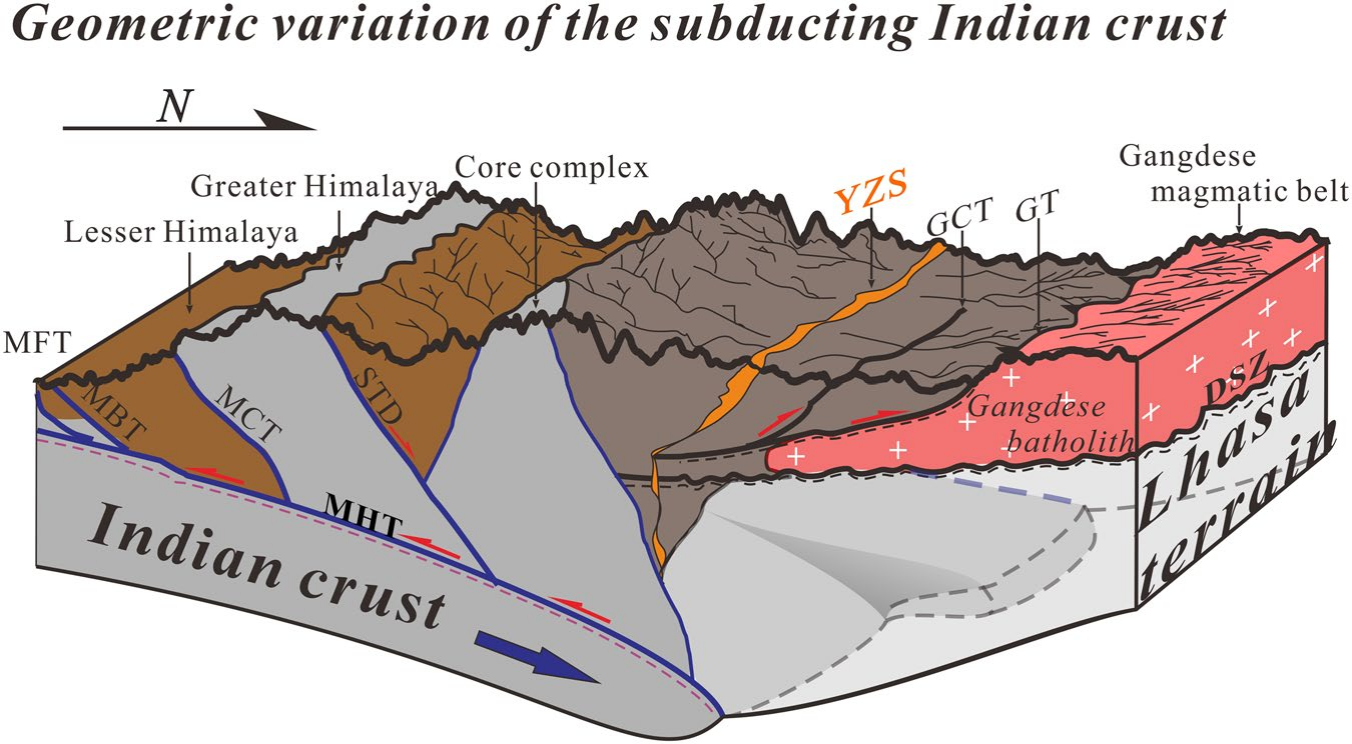
Interpretive sketch (not to scale) showing variation in the regional-scale crustal geometry of the Indian crust as it subducts beneath southern Tibet. Interpretations based on results from new deep seismic reflection images described here as well as previous seismic^8,14^ and structural studies^29^. MFT: Main Frontal Thrust; MBT: Main Boundary Thrust; MCT: Main Central Thrust; MHT: Main Himalayan Thrust; STD: Southern Tibetan Detachment; YZS: Yarlung-Zangbo suture; GT: Great Counter Thrust; GT: Gangdese Thrust. DSZ: Ductile shear zone; Figure was drafted by X.G. and X.X. using the software of CorelDRAW X5.

Figure 5 presents a sketch showing variation in the regional-scale geometry of the Indian crust as it subducts beneath southern Tibet. New high-resolution seismic reflection profiles described here reveal that India is underthrusting southern Tibet but only to a limited degree. When interpreted in combination with a previous profile of the western Himalayas, the new profiles indicate progressive eastward steepening and shortening, as well as only limited horizontal advance of the subducting Indian crust (Fig. 5). Our high-resolution seismic datasets provide a consistent image of crustal-scale geometry within the collision zone. Along with previous teleseismic receiver function studies^26^ and body wave tomographic studies^27^, new data presented here clearly demonstrate west to east spatial variation in the Indian lithosphere subducting beneath southern Tibet.

**Figure 6:**
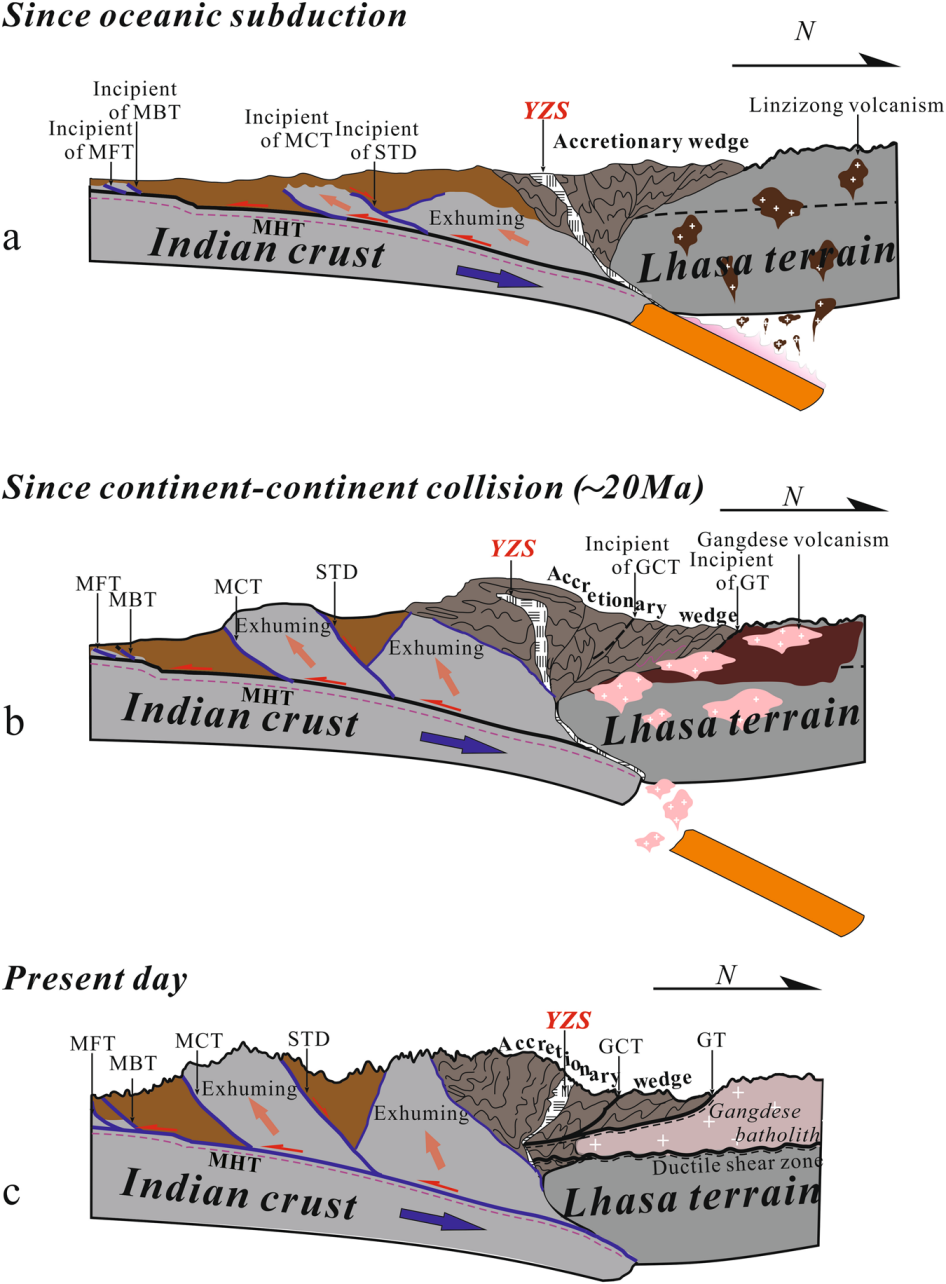
Block diagrams schematically illustrating a proposed crustal-scale tectonic evolution of the subducting Indian crust from (**a**) ocean-continent collision through (**b**) continent-continent collision to (**c**) present. Geometric variation of the contractional wedge as a result of rapid uplift of the northern Himalayas since ~20 Ma^28^ is highlighted. See discussion for details. MFT: Main Frontal Thrust; MBT: Main Boundary Thrust; MCT: Main Central Thrust; MHT: Main Himalayan Thrust; STD: Southern Tibetan Detachment; YZS: Yarlung-Zangbo suture; GT: Great Counter Thrust; GT: Gangdese Thrust. DSZ: Ductile shear zone; Figure was drafted by X.G. and X.X. using the software of CorelDRAW X 5.

Together with the deep seismic reflection profile in the western Himalayas, the results also outlined the limited thickness of Indian crust subduction beneath southern Tibet. We tentatively interpret this observation as evidence of continental materials resisting subduction due to crustal buoyancy. This process partly contributed to initiation of the Main Himalayan Thrust, which may act as an intracrustal ductile detachment fault decoupling deformation between the lower crust and overlying crust during continent-continent collision (Fig. 6a). As a result, the lower crust of the Indian plate was dragged beneath southern Tibet while the rest of the Indian crust peeled off and has experienced top-to-the-south duplexing or exhumation along the Main Himalayan Thrust to contribute to the rapid uplift of the Himalayan orogen^28^ (Fig. 6b). Consequently, an increase in the northward compressive forces occurred from the rapid uplift of the northern Himalayas^28^. The accretionary prism between the northern Himalayas and the Lhasa terrain experienced dramatic shortening and thickening (Fig. 6b). As a result, extensional collapse of the wedge under its own weight drove progressive development from the forewedge to the retrowedge. The ophiolitic suture zone experienced simultaneous reversal during this process and displays top-to-the north displacement bounding the ophiolitic mélange (Fig. 6c). On the other hand, contrasting material mechanics of the Tethyan sedimentary sequence and the relatively rigid Gangdese magmatic belt caused their differing responses to regional crustal shortening and rapid uplift of the northern Himalayas. They specifically experienced differing amounts of shortening while a detachment fault, the Gangdese thrust system (GT)^17^, developed along the margin between them (Fig. 6b and c). As northward compression continued, Tethyan sediment and the ophiolitic suture were thrusted atop the Gangdese magmatic belt.

Overall, the observations of both the limited extent of the Indian crust and lateral variation of the subduting Indian lithosphere require revision of interpretations regarding uplift and collapse of the Tibetan Plateau. Observations are specifically not consistent with a doubling of crustal thickness between the Indian crust and the Lhasa terrain evoked to explain the unusual crustal thickness of the Tibetan Plateau. Additionally, the timing and development history of lateral variation in the Indian lithosphere may contain important information for explaining sudden eastward plateau-wide collapse since mid-Miocene. Additional observation and interpretation is needed however in order to address this issue.

## Methods

### Data processing for producing the deep seismic reflection profile

Software modules from the CGG, Omega and GeoEast packages were used in seismic data processing. Processing procedures included static correction, true-amplitude recovery, frequency analysis, filter-parameter tests, surface consistent amplitude corrections, surface consistent deconvolution, coherent noise suppression, random noise attenuation, human-computer interactive velocity analysis, residual statics correction, Kirchhoff time migration for incorporating rugged topography, DMO stack and post-stack filtering to remove noise.

After fully analyzing and testing results of the raw data, targeted techniques were used to resolve several imaging problems. Tomographic static correction without ray tracing and multi-reflector interface residual statics were used to resolve static problems caused by irregular topographic relief and low-velocity structure of near surface layers. Pre-stack multi-domain processing was combined with noise attenuation techniques to suppress a range of noise sources. Human–computer interactive velocity analysis methods provided relatively accurate RMS (root-mean-square speed) estimates. Treatment of rugged topographic areas with Kirchhoff time migration improved the quality of seismic images. Four large dynamite shot gathers (charge ≥ 2000 kg) with high signal-noise ratios were processed to generate a single-fold profile. This profile revealed the main subsurface structures of the study area (Fig. 4 in the main text). The final migrated profile, contained abundant reflection events from the surface to the Moho discontinuity and provided a consistent image of the collision zone’s complex subsurface structure.
